
JOURNAL INFORMATION
==============================
NLM Title Abbreviation: ScientificWorldJournal
Journal ID: ScientificWorldJournal
NLM Journal ID: 101131163
Journal ID: TSWJ

The Scientific World Journal

ISSN: 2356-6140
EISSN: 1537-744X
Publisher: Wiley

ARTICLE INFORMATION
==============================
PMCID: PMC6084241
PMID: 30147613
DOI: 10.1100/tsw.2001.236
Article ID: 625323
Article version: 1
Subjects: Short Report

Single Step Fluorescent Caspase 3 Activity Assay: Quantifying the Inhibition of Cell Permeable Compounds in a Cell-Based System

Humpal-Winter Jean *
Niles Andrew
OBrien Martha
Bulleit Bob
Promega Corporation, 2800 Woods Hollow Road, Madison, WI 53711-5399, USA
*Jean Humpal-Winter: jhumpal@promega.com
Publication date: 2001
Electronic publication date: 2001 Nov 27
Volume: 1
First page: 135
Last page: 135
Copyright: Copyright © 2001 Jean Humpal-Winter et al.
Copyright year: 2001
License: This is an open access article distributed under the Creative Commons Attribution License, which permits unrestricted use, distribution, and reproduction in any medium, provided the original work is properly cited.
License URL: https://creativecommons.org/licenses/by/3.0/

==============================
Miami *Nature Biotechnology* Short Reports
*TheScientificWorld* (2001) 1 (S3), 135SR
ISSN 1532-2246; DOI 10.1100/tsw.2001.236

### SINGLE STEP FLUORESCENT CASPASE 3 ACTIVITY ASSAY: QUANTIFYING THE INHIBITION OF CELL PERMEABLE COMPOUNDS IN A CELL-BASED SYSTEM

Jean Humpal-Winter*, Andrew Niles, Martha O'Brien, and Bob Bulleit

Promega Corporation, 2800 Woods Hollow Road, Madison, WI 53711-5399, USA
* jhumpal@promega.com

**INTRODUCTION.** Caspases are a family of cysteine aspartyl proteases that are involved in the cleavage of key structural, cell signaling, and DNA repair substrates leading to apoptotic cell death (1). As a result, the modulation of caspase response is the object of intense study in clinical research. Caspase 3 is recognized as a pivotal effector and logical target for therapeutic intervention (2). To this end, we have developed a robust, highly sensitive, and rapid homogeneous caspase 3 assay that is quantifiable in cell-based assays and with purified active caspase 3. We have used this assay to monitor the efficiency of several cell-permeable caspase 3 inhibitors in the cell-based Jurkat/Fas-induction model system (3). The evaluation of these compounds by caspase activity was supported and correlated with the CaspACE$^{TM}$ FITC-VAD-FMK *in situ* cell imaging assay.

**METHOD.**
Ki determinations with purified active caspase 3: Purified active caspase 3 (PharMingen, USA) was used to determine the $Ki_{app}$ for the known and unknown inhibitors in our homogeneous caspase 3 assay. The procedure used was modified from the published methods of Garcia-Calvo et al. (4).

Apoptosis induction: Jurkat cells were grown to a density of $5.0 \times 10^5$ cells/ml in RPMI-1640 medium (HyClone, Logan, Utah). Cells were treated with 5.7μM Ac-DEVD-CHO (Promega Corporation, Madison, WI), 20μM Z-VAD-FMK (Promega Corporation, Madison, WI) inhibitors, and test compounds (UK1 and UK2) 30 minutes prior to apoptotic induction with Anti-Fas antibody (Pan Vera Corporation, Madison, WI), at 100ng/ml for 4 hours at 37°C and 5% $CO^2$. Control cells were treated with a comparable level of DMSO. After apoptotic induction, the cells were washed twice in PBS to remove excess inhibitor. The cells were resuspended in fresh RPMI-1640 medium and viability was determined by trypan blue exclusion. An aliquot from each treatment was labeled with CaspACE$^{TM}$ FITC-VAD-FMK (Promega Corporation, Madison, WI) for *in situ* visualization of caspase activity on a fluorescent microscope. The remaining cells were plated to 10,000 cells/well (100μl) in a 96 well Nunc Maxisorp white plate. Control wells for background readings did not contain cells. 100μl of homogeneous lysis buffer containing substrate was added to each well. The plate was incubated at room temperature on a shaker for about 1 hour prior to reading the fluorescence on a CytofluorII (PerSeptive Biosystems, Inc., Framingham, MA).

**RESULTS.** The cell permeable pan-caspase inhibitor Z-VAD-FMK and the sequence specific peptide inhibitor Ac-DEVD-CHO both inhibited the induction of apoptosis, as observed by the homogeneous caspase 3 assay and confirmed by fluorescence microscopy with CaspACE$^{TM}$ FITC-VAD-FMK. These results support the observations of Ac-DEVD-CHO inhibiting apoptosis in intact cells at high concentrations (5,6).

Apoptotic cells treated with UK1 and UK2 had higher caspase 3 activities and a greater percentage of FITC-VAD-FMK labeling than the apoptotic controls. Furthermore, UK2 treated cells had only 50% viability based on trypan blue exclusion after the 4-hour apoptotic induction, compared to 100% viability in the other treatment groups. These results suggest that doses of UK1 and UK2 that are three times the $Ki_{app}$ are toxic to the cells. We are unable to conclude if the UK1 and UK2 compounds are cell permeable due to the apparent toxicity. These results were surprising due to the observation that UK1 and UK2 inhibited purified caspase 3 in an *in vitro* assay.

**DISCUSSION.** The advent of high throughput screening techniques for chemical libraries has delivered many compounds active against the caspase-class proteases. Our studies emphasize that the active candidate compounds (UK1 and UK2) are not necessarily useful inhibitors upon careful secondary examination. The rapid and sensitive cell-based assay reported here is sufficiently flexible in format to determine relative Ki and cell permeability in order to quickly eliminate poorly efficacious compounds from lead compounds. This activity-based measure allows for critical dissection of a candidate compound's attributes (as caspase 3 agonist or antagonist) and ultimately allows for reduced drug discovery cycling times.

**REFERENCES.**

1. Earnshaw, W.C., Martians, L.M., and Kaufmann, S.A. (1999) Annu. Rev. Biochem. 383-424
2. Porter, A.G. and Janicke, R.U. (1999) Cell Death Differ. 6, 99-104
3. Yonehara, S., Ishii, A., and Yonehara, M. (1989) J. Exp. Med. 169, 1747-1756
4. Garcia-Calvo, M., Peterson, E.P., Rasper, D.M., Vallancourt, J.P., Zamboni, R., Nicholson, D.W., and Thornberry, N.A. (1999) Cell Death Differ. 6, 362-369
5. Nicholson, D.W., Ali, A., Thronberry, N.A., Vaillancourt, J.P., Ding, C.K., Gallant, M., Gareau, Y., Griffin, P.R., Labelle, M., Lazebnik, Y.A., Munday, N.A., Raji, S.M., Smulson, M.E., Yamin, T.T., Yu, V.L., and Miller, D.K. (1995) Nature 376, 37-43
6. Schlegel, J., Peters, I., Orrenius, S., Miller, D.K., Thronberry, N.A., Yamin, T.T., and Nicholson, D.W. (1995) J. Biol. Chem. 271(4), 1841-1844
